# Habitat properties and plant traits interact as drivers of non‐native plant species’ seed production at the local scale

**DOI:** 10.1002/ece3.3940

**Published:** 2018-04-02

**Authors:** Jonas J. Lembrechts, Evi Rossi, Ann Milbau, Ivan Nijs

**Affiliations:** ^1^ Centre of Excellence Plant and Vegetation Ecology University of Antwerp Wilrijk Belgium; ^2^ Openbare Vlaamse Afvalstoffenmaatschappij OVAM Mechelen Belgium; ^3^ Research Institute for Nature and Forest INBO Brussels Belgium

**Keywords:** alien plant species, invader success, invasibility, invasiveness, plant invasion, seed production

## Abstract

To answer the long‐standing question if we can predict plant invader success based on characteristics of the environment (invasibility) or the invasive species (invasiveness), or the combination of both, there is a need for detailed observational studies in which habitat properties, non‐native plant traits, and the resulting invader success are locally measured. In this study, we assess the interaction of gradients in the environmental and trait space on non‐native species fitness, expressed as seed production, for a set of 10 invasive and noninvasive non‐native species along a wide range of invaded sites in Flanders. In our multidimensional approach, most of the single environmental gradients (temperature, light availability, native plant species diversity, and soil fertility) and sets of non‐native plant traits (plant size, photosynthesis, and foliar chemical attributes) related positively with invader seed production. Yet correlation with seed production was much stronger when several environmental gradients were assessed in interaction, and even more so when we combined plant traits and habitat properties. The latter increased explanatory power of the models on average by 25% for invasive and by 7% for noninvasive species. Additionally, we report a 70‐fold higher seed production in invasive than in noninvasive species and fundamentally different correlations of seed production with plant traits and habitat properties in noninvasive versus invasive species. We conclude that locally measured traits and properties deserve much more attention than they currently get in invasion literature and thus encourage further studies combining this level of detail with the generality of a multiregion and multispecies approach across different stages of invasion.

## 1 INTRODUCTION

One of the fundamental and long‐standing questions in invasion ecology is if we can predict invasion success based either on characteristics of the environment (invasibility) or traits of the invasive species (invasiveness) (Hayes and Barry, 2008; Hui et al., 2016; Richardson & Pysek, 2006). Over the years, countless studies have sought patterns and generalities among the myriads of possible predictors that have been identified. Reviews, meta‐analyses, and large‐scale datamining approaches play an increasingly important role in this regard, by jointly assessing several possible drivers of invasion at once (Catford, Vesk, Richardson, & Pyšek, 2012; Chytry et al., 2009; Kueffer, Pysek, & Richardson, 2013; Lonsdale, 1999; Pysek, Jarosik, et al., 2009). Yet such large‐scale approaches, while highly valuable at the species level, often suffer from a reduced accuracy, as they rely on the aggregation of data from varying sources and are often limited to the use of approximated environmental or trait variables for which large datasets are available (Jansen, Ewald, & Zerbe, 2011; Jauni & Hyvonen, 2012; Milbau & Stout, 2008). Local observational studies, on the other hand, excel in detail and comparability of measurements, yet are often limited in the amount of explanatory variables assessed, measure only in a few sites, or consider only a few invasive species (e.g., Erfmeier & Bruelheide, 2010; Godefroid, Phartyal, Weyembergh, & Koedam, 2005). Additionally, habitat properties and plant traits are rarely combined into one study, while such an integration of species‐focused and environmentally focused research has been argued as one of the most important ways forward for invasion research (Kueffer et al., 2013; Milbau, Nijs, Van Peer, Reheul, & De Cauwer, 2003; Nijs, Milbau, & Seidlova, 2004; Richardson & Pysek, 2006).

While the combined study of invasiveness and invasibility has been limited, studies looking at one of these factors have identified several important drivers of plant invasion. In a meta‐analysis of plant traits associated with invasion, van Kleunen, Weber, and Fischer (2010) reported that invasive non‐native species tend to possess morphological or physiological characters that confer better performance (e.g., tall plants and large seed size (Crawley, Harvey, & Purvis, 1996; Zheng, Feng, Liu, & Liao, 2009) or high relative growth rates (Grotkopp, Rejmánek, & Rost, 2002; Grotkopp, Stoltenberg, Rejmánek, & Rost, 1998; Pattison, Goldstein, & Ares, 1998)). Invasive species also excel in nutrient‐use and photosynthetic efficiency, especially in nutrient‐poor environments (Funk, 2013; Funk & Vitousek, 2007; Leishman, Haslehurst, Ares, & Baruch, 2007; Pattison et al., 1998), and they often possess traits promoting rapid spread (Milbau & Stout, 2008). Yet many of the large‐scale studies of invasiveness encompassing several species in various environments have been limited to traits at the species level, neglecting variation in invasion success within species and across habitats.

Compared with identifying plant traits associated with invasiveness, habitat properties underlying invasibility have received significantly less attention (Catford et al., 2012). Still, invasion success is undeniably habitat‐dependent across all spatial scales (Carboni et al., 2016; Funk & Vitousek, 2007; Milbau, Stout, Graae, & Nijs, 2009). In those cases where invasibility is assessed, it is often carried out at a large scale and using rough habitat characteristics, for example, through the use of Ellenberg indicators (Carboni, Santoro, & Acosta, 2011; Chytry et al., 2009; Simonova & Lososova, 2008). The smaller scale, which is likely indispensable for accurate predictions of invader success, has been largely neglected (Lembrechts et al., 2017; Milbau et al., 2009). The available studies have linked plant invasions to higher temperatures, more mesic conditions, and higher nutrient levels in the soil (Burke & Grime, 1996; Rejmanek, Richardson, & Pysek, 2005; Simonova & Lososova, 2008), although the relative importance of these drivers has been shown to differ between environments (Richardson & Bond, 1991), and disturbance might overrule all of the above (Lembrechts et al., 2016; Rejmanek et al., 2005). Additionally, higher resident species diversity has often been associated with lower levels of invasion at the local scale (Knight & Reich, 2005; Levine, 2000), while across habitats, a higher diversity often tends to increase invasion levels (Lonsdale, 1999; Shea & Chesson, 2002; Stohlgren et al., 1999, 2002).

Despite the aforementioned advances in the assessments of invasiveness and invasibility, the combination of both has rarely been assessed (Drenovsky et al., 2012; Richardson & Pysek, 2006). Vicente, Alves, Randin, Guisan, and Honrado (2010) and Vicente et al. (2013) did, however, report variable responses to environmental gradients for plants with different ecological strategies, while ecological resistance has been shown to be overwhelmed by a high propagule pressure (Von Holle & Simberloff, 2005). Additionally, Carboni et al. (2016) described how the crucial role of habitat characteristics on a coarse scale in driving plant invasions got supplemented with important effects of species‐specific traits at the finest scale. Invasiveness and invasibility thus likely interact with each other, with some ecosystems being more (or only) invasible by species with certain traits (Funk & Vitousek, 2007). Pinpointing these interactions requires extensive measurements of both habitat properties and plant traits at many sites at the local scale.

The literature on biological invasions has been biased in favor of invasive species—those that spread vigorously and often reach high abundance following introduction by humans (Richardson & Pysek, 2012). It is however highly relevant to also understand the previous stages in the invasion process, especially the factors that mediate naturalization (see for instance Milbau & Stout, 2008). The emphasis on successful invaders partly originates from the fact that most invasions are only recognized once species invaded significantly large areas, or start having an impact on the environment. Also, many studies lump all non‐native species and fail to separate introduced, naturalized, and invasive species (Richardson & Pysek, 2012). These biases limit our ability to identify the full set of drivers of invasion, as different factors might mediate non‐native species performance at different stages of the invasion continuum (Dietz and Edwards (2006); Pysek et al. (2008), but see Smith and Knapp (2001)).

The invasion process is clearly complex, and extrapolation of invasiveness of a species from one system to another might be inadvisable. Kuster, Kuhn, Bruelheide, and Klotz (2008) and Kueffer et al. (2013) therefore suggest that invasion ecology research should be conducted on multiple sites and by integrating a wide range of factors and their interactions to detect general patterns of invasion. In this study, we combined an unprecedented amount of detailed measurements on plant traits and habitat properties at the population scale along a large set of invaded sites in Flanders, for 10 invasive and noninvasive non‐native species. Along these gradients of variation in environmental conditions and plant traits, we assessed the relative role of invasiveness and invasibility in non‐native species fitness, expressed as seed production. On the basis of the premise that several of the studied environmental gradients (temperature, light availability, soil fertility, and native species diversity) and sets of non‐native plant traits (photosynthesis, plant size, and foliar chemical attributes) have already been identified as key drivers of invasion success in previous studies, we expect positive effects of all of them, yet hypothesize that explanatory power will increase significantly when we allow habitat properties and plant traits to interact with each other in the statistical models. The direction and importance of each factor and interaction might differ between invasive and noninvasive species.

## 2 MATERIAL AND METHODS

### 2.1 Design

We studied 10 species non‐native to Belgium, half of them highly invasive and the other half naturalized but not (yet) invasive at the moment of the survey (Verloove, 2002). Species status was identified using the definition and classification of non‐native plants for Flanders from Verloove (2002), who followed the definition from Richardson et al. (2000). To be considered invasive, a non‐native species had to (1) spread rapidly (>2 m/year for vegetative reproduction, >100 m for generative reproduction), (2) colonize the anthropogenic environment, and (3) penetrate the (semi‐)natural environment. *Fallopia japonica* (Houtt.) Ronse Decraene (Japanese Knotweed, Eastern Asia), *Heracleum mantegazzianum* Somm. et Lev. (Giant Hogweed, Southwest Asia), *Impatiens glandulifera* Royle (Himalayan Balsam, Himalaya), *Senecio inaequidens* DC. (South African ragwort, South Africa), and *Solidago gigantea* Ait. (Giant Goldenrod, North America) were invasive (English name and native range between brackets), whereas *Cerastium tomentosum* L. (Snow‐in‐summer, Italy), *Impatiens parviflora* DC. (Small Balsam, Middle and Eastern Asia), *Lathyrus latifolius* L. (Perennial Sweet Pea, South, Middle and Eastern Europe), *Rosa rugosa* Thunb. (Rugosa Rose, Eastern Asia), and *Xanthium orientale* L. (Californian Burr, America) were noninvasive. For *S*.* gigantea*, the status was updated from established (as mentioned by Verloove, 2002) to invasive based on local work at the moment of the survey from Vanderhoeven, Dassonville, Chapuis‐Lardy, Hayez, and Meerts (2006). The average year of introduction to Belgium was 1913 and 1899 for invasive and noninvasive species, respectively (Verloove, 2002), minimizing the effect of residence time on invasion success. Different life forms and growth habits were included in order to detect possible relationships between traits and seed production that are sufficiently widely valid.

**Figure 1:**
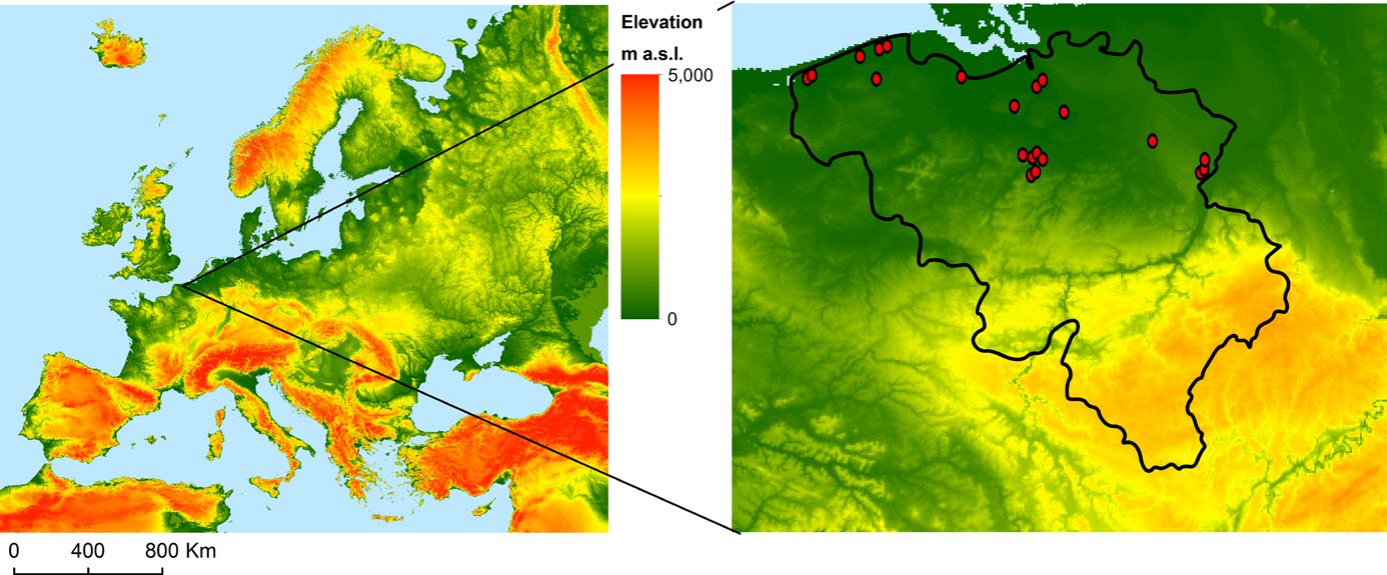
Observational field sites (red dots, right) within the study region in the northern part of Belgium and its location within Europe (left)

Each of the 10 species was studied at three sites across Flanders, yielding 30 sites in total (Figure 1), 13 of which were situated in nature reserves. Divergent sites per species (e.g., woodland, grassland, roadside verges) were chosen to have a wide range of habitat properties. At each site, we quantified (1) properties of the invaded habitat on a distance of a few meters from the patch of the non‐native species and (2) traits of the non‐native population within the patch. Traits and properties were selected taking into account that reproductive output relates to resource uptake and supply, (internal re‐)allocation of resources, plant physiology and morphology, environmental characteristics, biotic interactions, and soil fertility (Bazzaz, Ackerly, & Reekie, 2000). When relevant, traits and properties were sampled in spring (26 May–27 June 2003) and in summer (21 July–7 August 2003). Two seasons were included to ensure detection of traits and properties that are only of influence in one season. Non‐native fitness itself was expressed as total seed production per plant, measured in 10 reproducing individuals per site. Even though not all studied species rely solely on seeds for their reproduction, seed production is a trustworthy predictor of an individuals’ fitness, as it provides the culmination of all traits and environmental characteristics throughout the whole life cycle (Williamson & Fitter, 1996), and part of the variation in seed production in the data set originates from within‐species variation (i.e., across habitats). The reader should, however, keep in mind that invader fitness might in some cases not be fully correlated with the measured seed production.

### 2.2 Measurements

#### 2.2.1 Properties of the invaded habitat

Properties of the invaded habitat were measured in the vegetation surrounding the invaders, to avoid possible modification of habitat characteristics by the non‐native species. No apparent dissimilarity between adjoining invaded and uninvaded plots was allowed (e.g., slope and elevation), other than invader presence. At each site, we measured the following habitat properties (summarized in Table S1):

1. *Temperature*. Air temperature (*T*, °C) was measured with a Kestrel 3000 Pocket Weather Meter (Nielsen‐Kellerman, Boothwyn, PA, USA), yielding temperature below and above the non‐native species canopy, and their difference (Δ*T*, below minus above the canopy). All temperature measurements related positively with principal component analysis (PCA) axis 1 (Figure S1a, Table S1).
2. *Light*. Photon flux density of photosynthetically active radiation was determined with a JYP 1000 gallium arsenide quantum sensor (SDEC, Reignac‐sur‐Indre, France), yielding absolute PAR availability above and below the vegetation, as well as PAR penetration (PAR ratio, ground level/top of canopy). Almost all light measurements related negatively with PCA axis 1, with the strongest negative correlation for the PAR ratio (Figure S1b, Table S1).

Both temperature and light were sampled as seasonal properties (spring and summer) as the average of observations at five replicate locations at ground level (so as experienced by alien seedlings) and at the top of the canopy to quantify how they were modified by the vegetation.

1. *Soil nutrients*. In each of six randomly selected 1‐m² plots within each site, five soil cores were sampled (0–10 cm depth, 4 cm diameter, once per location between February and April), pooled, and homogenized. Soil samples were air‐dried until constant mass and sieved (<0.2 cm). The following parameters were assessed: soil pH (stiff paste soil‐H_2_O, pH_H2O_, and stiff paste soil‐KCl, pH_KCl_) and trace elements (ICP‐AES determination of Ca, Cu, K, Mg, Mn, P, and Zn, g/kg). Total C and N were assessed with a dry combustion C/N analyzer (C_tot_, N_tot_, g/kg). Carbon in CaCO_3_ (Ccarbo) was assessed after calcination of organic matter at 450°C (dry combustion, Ströhlein dosimeter). Organic C (Corg) was calculated as Ctot‐Ccarbo. Soil nutrient concentrations in general (and carbon‐ and nitrogen‐concentrations in particular most strongly) related negatively with PCA axis 1 (Figure S1c, Table S1).
2. *Plant diversity*. Plant species diversity in the surrounding vegetation (thus natives only) was estimated once (July–August): At every site, species diversity was measured in 1‐m^2^ plots, constructing a saturating curve by adding additional 1‐m² plots until no more new species were recorded, yielding the total species richness (*R*) of the site. Species diversity was calculated for each of these plots with the Shannon index $H=-\sum_{i=1}^{N}a_{i}\;\text{ln}\;a_{i}$, with *a*_*i*_ the relative species cover and *N* the number of species in the plot. The average *H* value of all plots in a site yielded the property value for that site. The Pielou evenness index $J=-\left(\sum_{i=1}^{N}\;a_{i}\;\;\text{ln}\;\;a_{i}\right)/\text{ln}\;N$ was likewise computed per plot and averaged. All diversity measures related negatively with PCA axis 1 (Figure S1d, Table S1).

#### 2.2.2 Traits of non‐native species

Because most plant traits are dependent on environmental conditions, we measured them in the field and not in a greenhouse in pots. To allow for the possible influence of native competitors, traits were determined on isolated non‐native plants near the front of the non‐native population, rather than in the center of non‐native monocultures. For each species at each site, we measured the following plant traits (summarized in Table S2):

1. *Photosynthesis*. This was determined on the youngest fully expanded leaf (YFEL) of four replicate plants, both in spring and summer, and provided an indication of the photosynthetic abilities of the study plants. We measured light‐saturated photosynthetic rate (*P*_max_, μmol CO_2_ m^−2^ s^−1^) and apparent quantum efficiency (α, μmol CO_2_/μmol photons) as a measure of photosynthetic efficiency and dark respiration rate (*R*_d_, μmol CO_2_ m^−2^ s^−1^) and light compensation point (PARc, μmol photons m^−2^ s^−1^) as a measure of adaptation to shade (Givnish, Montgomery, & Goldstein, 2004). These variables were derived from CO_2_ exchange rates at four light intensities: 1,800 or 1,200 (the latter for shaded sites), 100, 50, and 0 μmol photons m^−2^ s^−1^. Readings were taken with a LI‐6400 gas exchange system (LI‐COR, Inc., Lincoln, NE, USA) at ambient humidity and leaf temperature between 20 and 25°C. Light intensities were decreased on the same leaf, with minimum 4‐min stabilization time in between. The reported *P*_max_ and *R*_d_ values are the measured rates at 1,800 (1,200) and 0 μmol photons m^−2^ s^−1^, respectively, and α and PARc are the calculated slope and X‐intercept of the straight line connecting the values at 0 and 50 μmol photons m^−2^ s^−1^. Additionally, after analyzing leaf nitrogen (NYFEL) with a dry combustion C/N analyzer (NC‐2100; Carlo Erba Instruments, Milan, Italy), instantaneous photosynthetic nitrogen use efficiency (PNUE, μmol CO_2_ mol N^−1^ s^−1^) was calculated as Pmax.NYFEL.M_N_^−1^ with M_N_ the atomic mass of nitrogen. Nearly all of these measurements related positively with axis 1 of the PCA of photosynthesis (Figure S1e, Table S2).
2. *Plant size*. Both in spring and summer, five randomly chosen, solitary non‐native plants were harvested above and belowground and dried (75°C, 24 hr), after measuring their height (cm). Subsequently, each plant was divided into root biomass (Br, g), leaf blades (Bl, g), and other aboveground material (Bo, g) to calculate shoot biomass as Bs = Bl + Bo (g), total biomass as Bt = Br + Bl + Bo (g), root: shoot ratio as Br/Bs, and leaf mass ratio (LMR) as Bl/Bt. Specific leaf area (SLA) was measured on each YFEL used above for photosynthetic measures, by taking the leaf area and the weight of each leaf or leaf part analyzed with the LI‐COR. Plant size measurements related negatively with axis 1 of their PCA, except for LMR and SLA (Figure S1f, Table S2).
3. *Foliar chemical attributes*. To measure leaf chemical attributes, the leaf part used to determine CO_2_ exchange rate was excised to measure the leaf area (*A*_leaf_, cm^2^) with a leaf scanner, then dried (75°C, 24 hr), weighed (*B*_leaf_, g), and analyzed for carbon and nitrogen concentration on a mass basis (C, N, g/kg) with a dry combustion C/N analyzer (NC‐2100; Carlo Erba Instruments). Carbon‐to‐nitrogen ratio (C/N, g/g) was then calculated as C×N^−1^. A separate series of YFEL (10 replicates, each on a different plant) were harvested at the beginning of the growing season (May–June, depending on species), dried (75°C, 24 hr), and analyzed with ICP‐AES for mineral nutrients (Ca, Cu, Fe, K, Mg, Mn, P, and Zn, g/kg). Most chemical leaf attributes (except carbon‐ and nitrogen‐concentrations) related negatively, and C/N‐ratios positively, with PCA axis 1 (Figure S1g, Table S2).

### 2.3 Statistical analyses

We used a combined PCA‐regression approach adapted from Jolliffe (2002) and Zuur, Ieno, and Smith (2007) to reduce multidimensionality in our multivariate dataset. Each category of environmental properties (properties related to temperature, light, soil characteristics, and native plant diversity) and non‐native plant traits (traits related to photosynthesis, plant size, and foliar chemical attributes) was reduced to one gradient with a PCA (function *dudi.pca* from R package ade4). From each of these seven PCAs, we extracted the loadings of the first axis for further analysis (Table 1) as well as the principal components (i.e., the coordinates), visualized in Figure S1 and listed in Tables S1 and S2. With this approach, we could reduce different related traits or properties to one dimension covering the largest part of the variation and as such increase interpretability of our multivariate dataset. While this reduces the level of detail, the strong collinearity between most variables within each group of traits and properties suggests we cover the most relevant gradients with our approach (Figure S1).

**Table 1:** Overall gradients covered by axis 1 of the PCA (with percentage of variance explained) for each category of habitat properties (top) and plant traits (bottom)

Category	PCA axis 1	% of variance explained
Temperature	Lower‐to‐higher temperatures	40.34
Light	Higher‐to‐lower light availability	54.45
Soil nutrients	Higher‐to‐lower C and N concentrations	26.73
Diversity	Higher‐to‐lower diversity	75.53
Photosynthesis	Lower‐to‐higher photosynthesis	37.90
Plant size	Larger‐to‐smaller plants Higher‐to‐lower biomass Lower‐to‐higher SLA and LMR	26.57
Foliar chemical attributes	More‐to‐less trace elements (especially Cu, P, Ca, Mg, K, Zn, and Fe) and lower‐to‐higher C/N‐ratio	36.09

PCA, Principal component analysis.

To explain differences in seed production (ln(seed production + 1)), we constructed several linear models containing various combinations of the explanatory variables (i.e., the first axes of their respective PCAs) to assess the explanatory power of (1) all single sets of traits and properties, versus models with interactions either (2) within or (3) between the plant traits and habitat properties.

The constructed models thus include:

1. A first model with only habitat properties and plant traits without interactions.
2. Two separate models with either all groups of habitat properties or all groups of plant traits and with all their two‐way interactions.
3. Three models including the interaction between one set of species traits and all habitat properties.

Note that it was not possible to combine the latter three models into one model containing all plant traits and habitat properties and their interactions, due to overfitting of the data in such a model. By making three separate models to assess the interaction of each plant trait with all habitat properties, the amount of models was kept to the lowest possible level. We used a Bonferroni correction to adjust for multiple testing, by multiplying each *p*‐value with the number of tests.

Explanatory power of the different models was assessed by comparing their marginal *R* ^2^, for noninvasive and invasive species separately. In all these models, ln(seed production + 1) was assessed with a linear mixed model (function *lme* from R package lme4), with a random structure with plot nested into species. The random structure was optimized through a likelihood test (function *ANOVA* in R) comparing the full model with models containing only plot or species as a random effect. Subsequently, the fixed structure was optimized by step‐by‐step removing the variable with the highest *p*‐value in models fitted with a maximum‐likelihood method and assessing the effect of each omitted variable with a likelihood test. Use of the random structure of plot nested in species was only necessary in the model without environmental factors; in all other models, we only needed to take species identity into account. When possible, species status (invasive/noninvasive) and its two‐way interactions with all explanatory variables were included in the models. In the third set of models, however, noninvasive and invasive species were analyzed in different models due to the risk of overfitting in the full model. All these analyses were performed across all species, driven by our aim to identify overarching traits and properties driving seed production and invader fitness (Nijs et al., 2004).

All data analyses were performed in R 3.0.1 (R Core Team, 2015).

## 3 RESULTS

**Figure 2:**
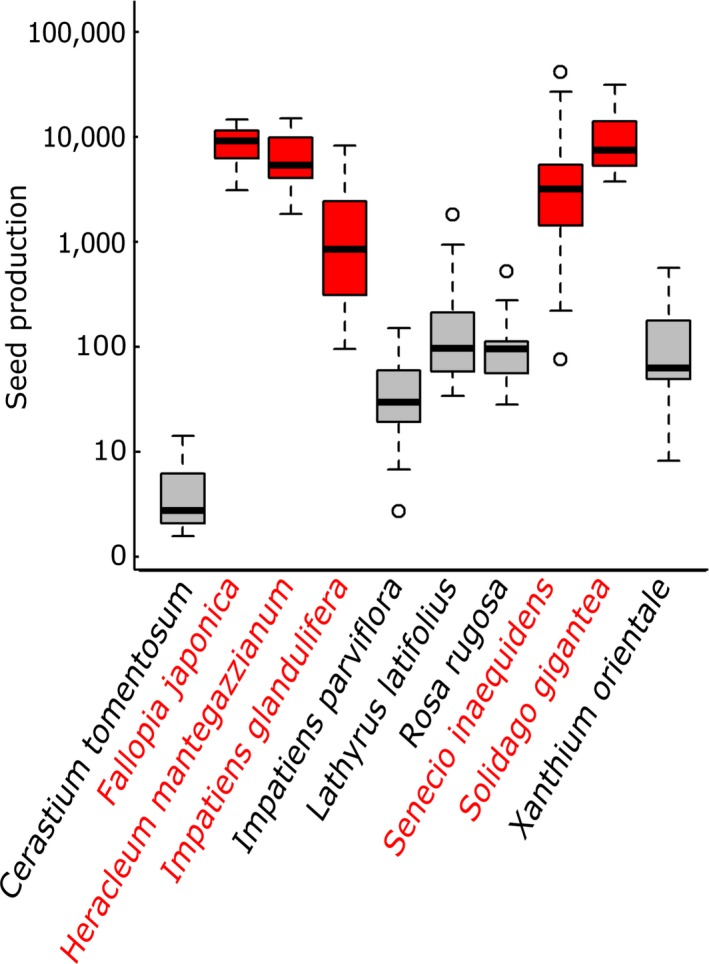
Boxplots of the logarithm of seed production for each of the noninvasive (black) and invasive (red) study species

**Figure 3:**
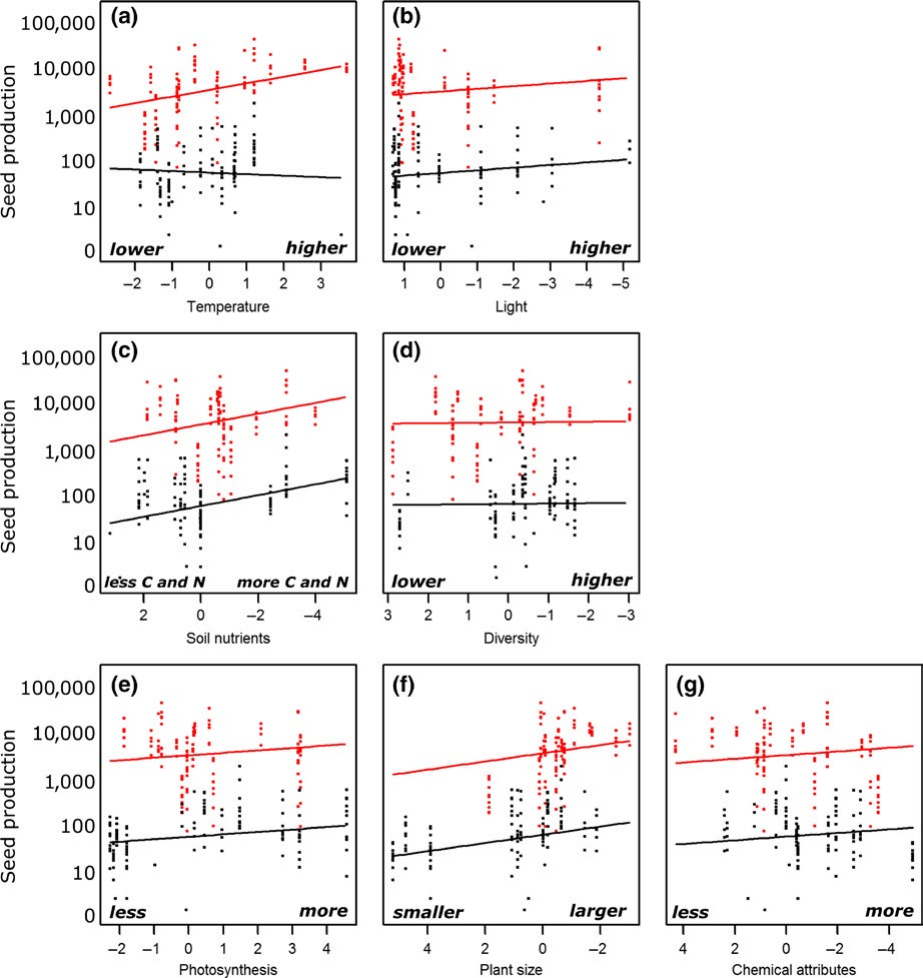
Unidimensional representations of the multifactorial linear mixed model for the logarithm of seed production as a function of the first axis of all PCAs of habitat properties (a–d) and plant traits (e–g) without interactions, for noninvasive (black) and invasive (red) species. Dots represent individual seed production. Note that several axes are reversed to increase interpretability. For the full model, see Table S3. PCA, Principal component analysis

Seed production of non‐native species related most strongly to species status: Invasive non‐native species produced on average approximately 70 times more seeds than noninvasive non‐natives (Figure 2), yet both species groups related similarly to most singular plant traits (photosynthesis, plant size, and foliar chemical attributes) and habitat properties (diversity, light, and soil fertility, yet differently to temperature) (Figure 3, Table S3).

**Figure 4:**
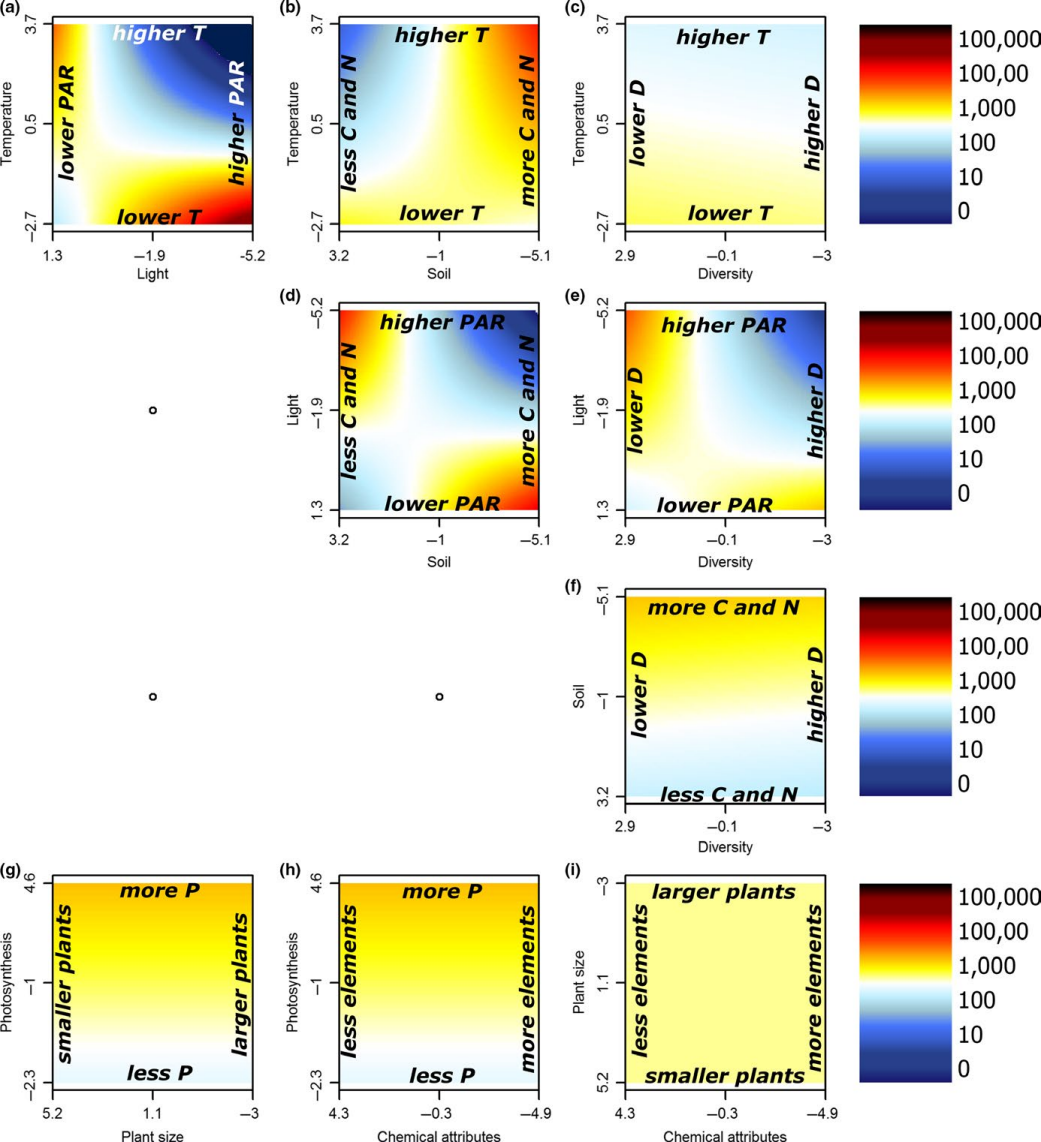
Two‐dimensional representations of the linear mixed models for the logarithm of seed production for the invasive non‐native species as a function of the first axes of all PCAs. Two separate models were constructed for the habitat properties (a–f) and plant traits (g–i) and their respective two‐way interactions. Each panel shows the interaction between two of the traits and properties. Red colors indicate positive (and blue negative) deviations from average seed production. For the full models, see Table S4. Note that several axes are reversed to increase interpretability. PCA, Principal component analysis

**Figure 5:**
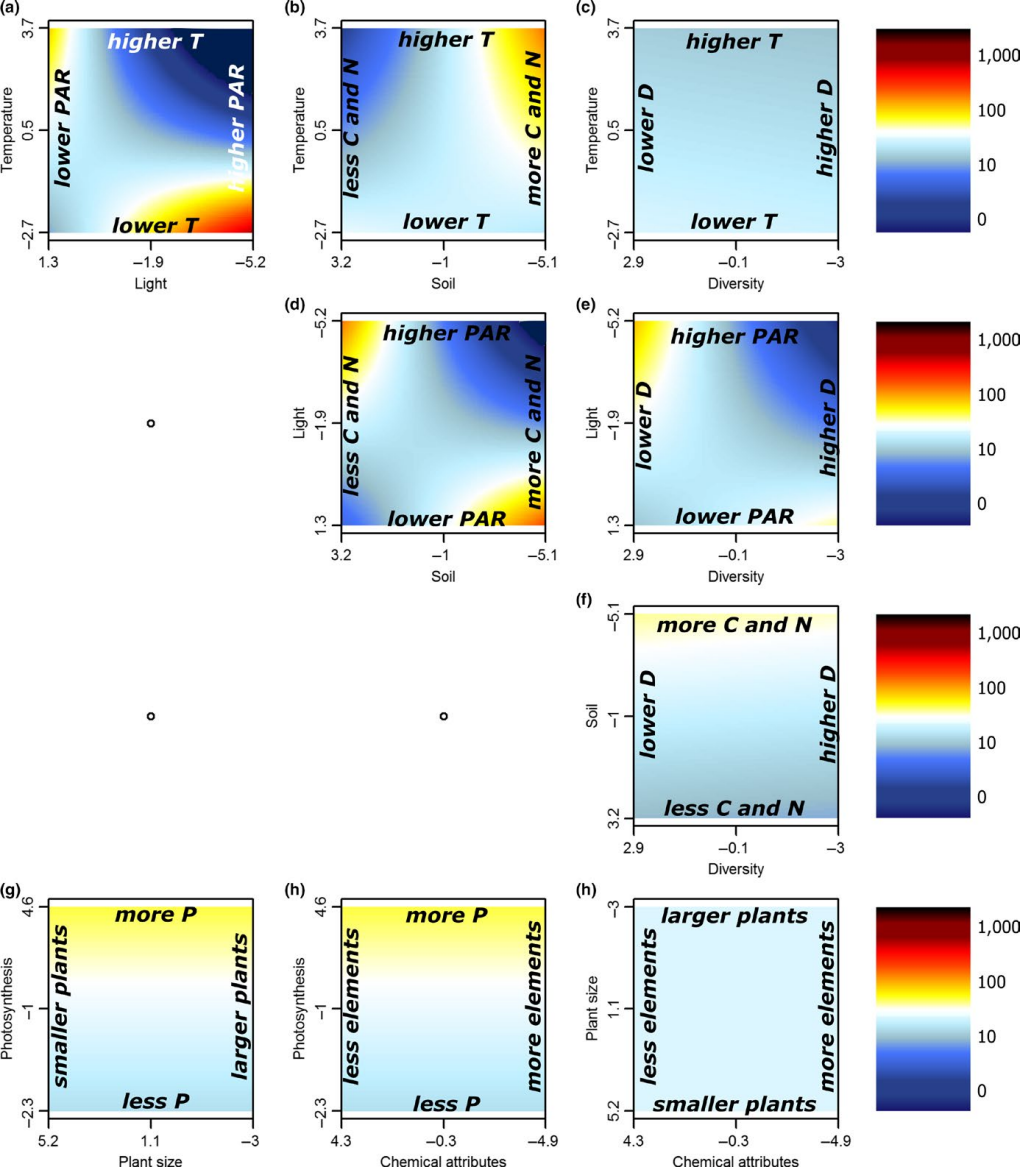
Two‐dimensional representation of the linear mixed models for the logarithm of seed production for the noninvasive non‐native species as a function of the first axis of all PCAs of habitat properties (a–f) and plant traits (g–i). Two separate models were constructed for the habitat properties (a–f) and plant traits (g–i) and their respective two‐way interactions. Each panel shows the interaction between two of the traits or properties. Red colors indicate positive (and blue negative) deviations from average seed production. For the full models, see Table S4. Note that several axes are reversed to increase interpretability. PCA, Principal component analysis

When taking into account interactions between environmental variables, a higher diversity in patterns was observed. In warmer environments, invasive species produced more seeds in low light than in high light conditions (Figure 4a, Table S4) or in C‐ and N‐rich than in C‐ and N‐poor soils (Figure 4b). In cooler environments however, invasive species produced more seeds in high than in low light conditions, while there was no effect of soil nutrient concentrations (Figure 4a,b). Similarly, richer soils or plots with high plant diversity gave highest seed production in low light conditions (Figure 4d,e), yet in poorer soils or plots with low diversity, high light availability still boosted seed production (Figure 4d,e). Noninvasive species showed similar relationships with habitat properties (Figure 5), yet they additionally produced relatively more seeds in warmer plots with a low diversity (Figure 5c). Plant traits did not show any significant interactions with each other, and in the model without any habitat property, only photosynthesis turned out (borderline) significant (Figures 4 and 5g,i, Table S4). Species status was the single important explanatory variable in this case.

**Figure 6:**
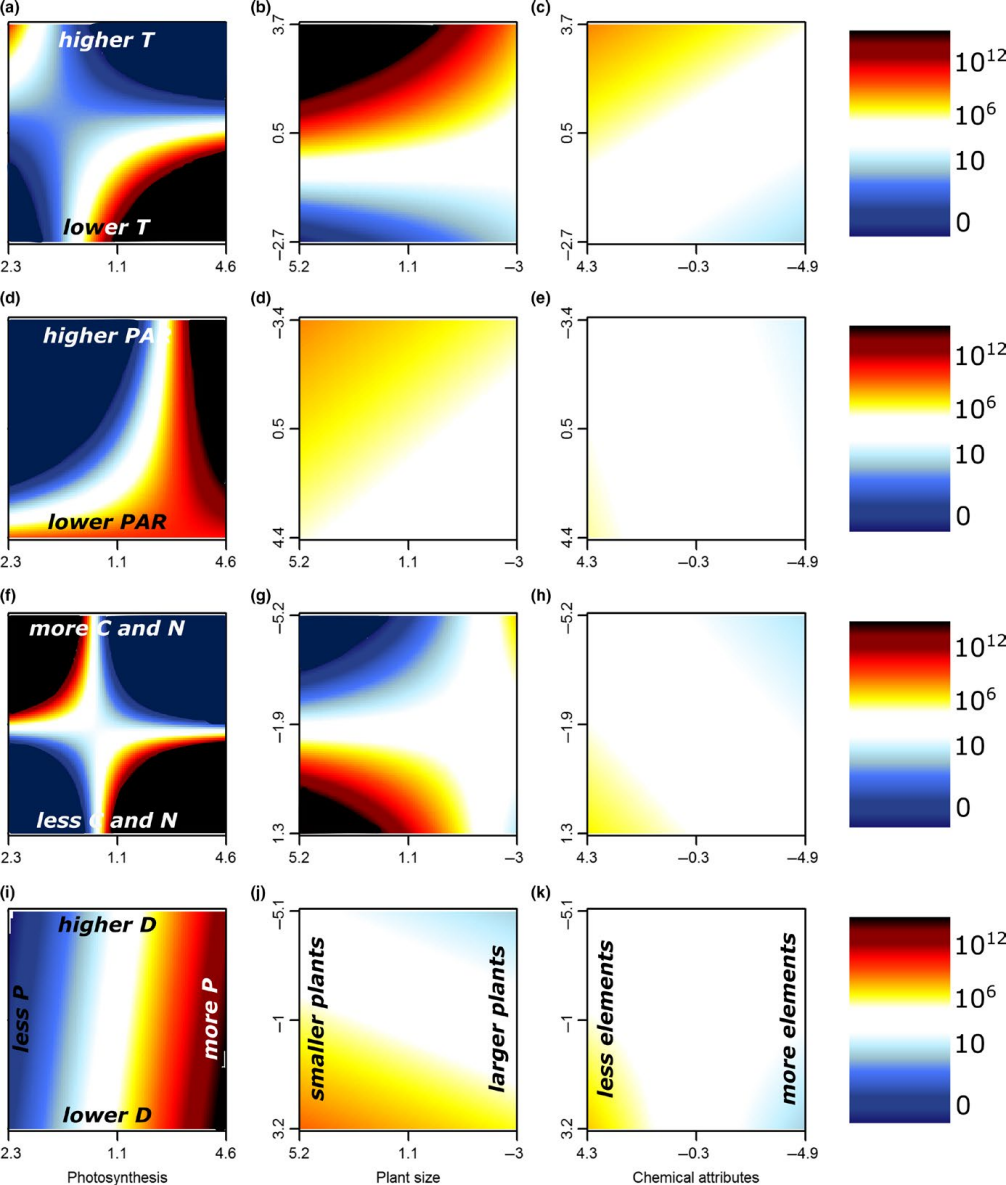
Two‐dimensional representation of the optimal linear mixed models for the logarithm of seed production for the invasive non‐native species as a function of the interactions of the first axes of all PCAs of habitat properties (*y*‐axis) and plant traits (*x*‐axis). Each panel shows the interaction between one trait and property. Red colors indicate positive (and blue negative) deviations from average seed production. For the full models, see Table S5. Note that several axes are reversed to increase interpretability. PCA, Principal component analysis

**Figure 7:**
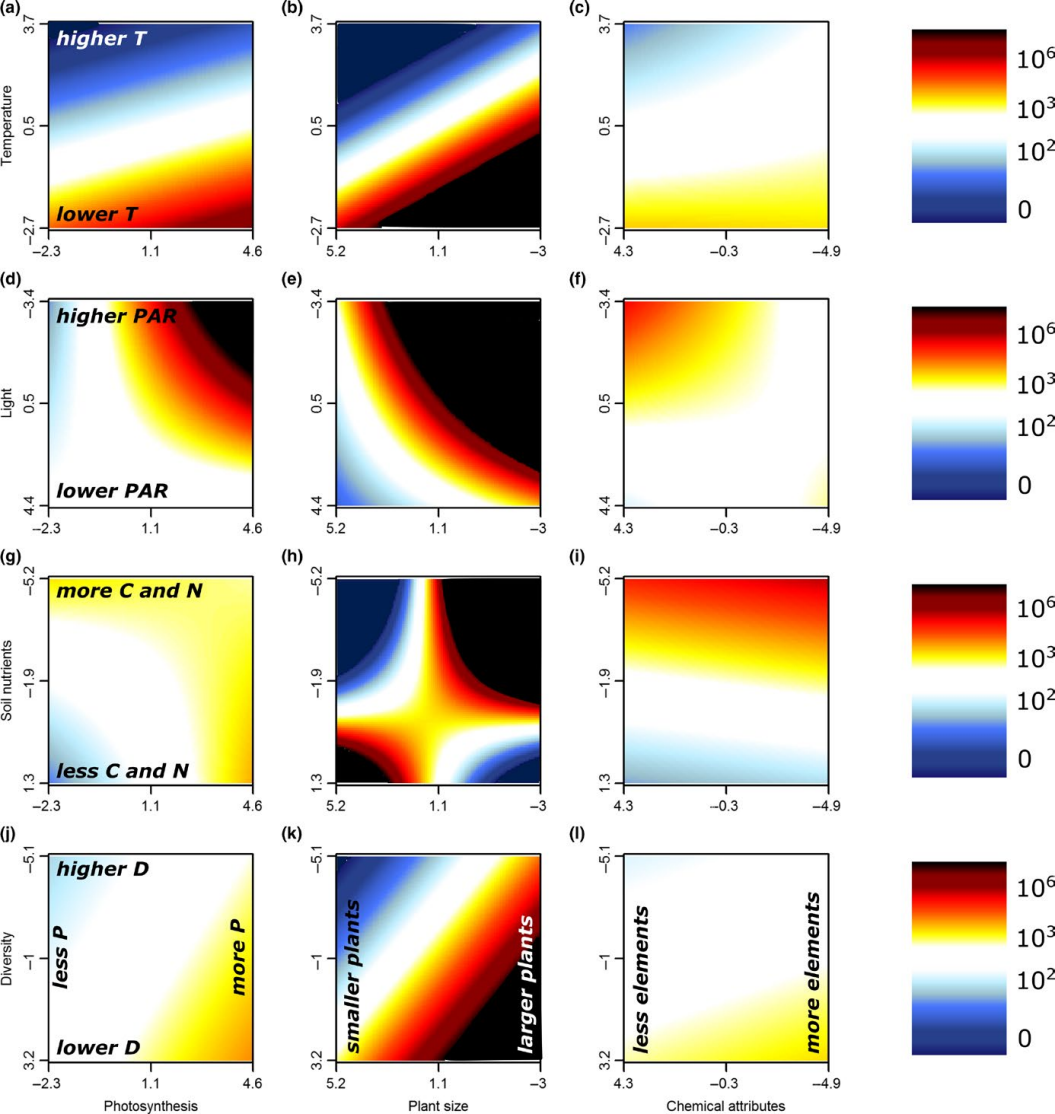
Two‐dimensional representation of the optimal linear mixed models for the logarithm of seed production for the noninvasive non‐native species as a function of the interaction of the first axes of all PCAs of habitat properties (*y*‐axis) and plant traits (*x*‐axis). Each panel shows the interaction between one trait and property. Red colors indicate positive (and blue negative) deviations from average seed production. For the full models, see Table S5. Note that several axes are reversed to increase interpretability. PCA, Principal component analysis

Even though plant traits on their own played a limited role in explaining seed production, we did find strong support for multiple additive effects and/or significant interactions between plant traits and environmental gradients (Figures 6 and 7, Table S5). Photosynthetic efficiency of the invasive populations, for example, turned out an important driver of invader seed production in habitats with lower temperatures, higher PAR, and poorer soils (Figure 6 first column). At the same time, lower levels of photosynthesis efficiency had a positive effect in plots with richer soils. While larger plants in general produced more seeds (Figure 3), smaller‐sized invader populations produced more seeds in warmer or lighter plots, poorer soils, and plots with a lower diversity (Figure 6 second column). Invader foliar chemical attributes did not interact strongly with environmental conditions, yet lower trace element concentrations resulted in slightly higher seed production in plots with higher temperatures, lower soil nutrient richness, and lower native species diversity (Figure 6 third column). For noninvasive species, the environmental variables interacted more strongly with plant size than with photosynthesis and foliar chemical attributes; we observed highest seed production for noninvasive populations with larger plants in colder plots, or plots with high light availability, rich soils, or low diversity (Figure 7 second column).

The interaction between habitat properties and plant traits (Table 2 part (3)) increased model explanatory power on average by 25% for invasive and 7% for noninvasive species compared to the models without these interactions (Table 2, part (1) and (2)). Plant traits explained limited variance on their own (16%), yet modeling them in interaction with environmental properties increased the explained variance by an average of 23% (Table 2).

**Table 2:** Marginal *R* ^2^ for the linear mixed models of the logarithm of seed production with either no interactions (1), plant traits, or habitat properties separately (2), or the interaction between traits and properties (3), for invasive (top) and noninvasive (bottom) species separately

(1)	(2)		(3)		
Single factors	Plant traits	Habitat properties	Photosynthesis × Habitat	Plant size × Habitat	Foliar chemical attributes × Habitat
0.281	0.066	0.196	0.422	0.347	0.535
0.271	0.256	0.276	0.402	0.285	0.330

## 4 DISCUSSION

In our multivariate approach, most single habitat properties and plant traits related positively, albeit sometimes weakly, to invader seed production (Figure 3), consistent with the patterns most commonly observed in the literature. Temperature (Lembrechts et al., 2016; Richardson & Bond, 1991; Stohlgren et al., 2002), light availability (Knight, Oleksyn, Jagodzinski, Reich, & Kasprowicz, 2008; Milbau, Nijs, De Raedemaecker, Reheul, & De Cauwer, 2005), soil fertility (Simonova & Lososova, 2008; Stohlgren et al., 2002), photosynthetic efficiency (Feng, Fu, & Zheng, 2008; Godoy, Valladares, & Castro‐Diez, 2012; Zheng et al., 2009), and non‐native plant size (Godoy et al., 2012; van Kleunen et al., 2010; Zheng et al., 2009) have indeed all been shown to be relevant drivers of invasion success in many studies at different spatial scales. Even the combined positive effects of several of these habitat properties (like temperature and soil fertility) and plant traits (like photosynthesis and plant size) have been shown before (Godoy et al., 2012; Jansen et al., 2011; Lembrechts et al., 2017; Simonova & Lososova, 2008; Zheng et al., 2009). Yet our study supports our hypothesis that it is vital to include interactions between habitat properties and plant traits in the assessment of invader success, as some plant traits can have positive effects in some and negative effects in other environments. Our data indeed showed on average a 23% increase in the explanatory power of plant traits at the population scale when assessed in interaction with the local environment (Table 2).

Our ability to observe interactions between habitat properties and plant traits heavily relied on the local nature of our approach. Many existing large‐scale studies indeed mostly cover broad habitat characteristics (Chytry et al., 2008; Pysek, Jarosik, et al., 2009; yet see Stohlgren et al., 2002), use environmental data with a large grain size (Petitpierre et al., 2012), approximate environmental conditions through plant traits like Ellenberg values (Simonova & Lososova, 2008), or only assess plant traits at the species level (Carboni et al., 2016; Jauni & Hyvonen, 2012; Vicente et al., 2010, 2013). Yet we do know that different drivers of invasibility (like biotic interactions, disturbance, and soil types on top of climate and land use) might be relevant at smaller spatial scales (Milbau et al., 2009).

Plant traits indeed related more strongly to invader seed production in our study when modeled with than without acknowledging the environmental variation. Even though most large‐scale assessments of invasion success at the species level have shown positive effects of the plant traits covered here (Rejmanek & Richardson, 1996), our interactive approach highlights that such plant traits in general have limited predictive power on the local scale, without taking into account the characteristics of the specific habitat (Pysek et al., 2015). For example, it has been shown before that invaders tend to maximize photosynthesis in nutrient‐limited soils (Matzek, 2011). This correlation is in accordance with our observation of increased invader seed production with higher levels of photosynthetic efficiency in resource‐poor soils (Figure 6g). Additionally, we showed a positive effect of the interaction between photosynthetic efficiency and lower environmental temperatures on seed production in both noninvasive and invasive species (Figures 6 and 7a). These results support the recent theory that invasive species might also be able to invade successfully in resource‐poor environments, when their traits are adapted accordingly (Funk, 2013; Funk & Vitousek, 2007; Schumacher, Kueffer, Edwards, & Dietz, 2009), an observation easily overlooked in large‐scale assessments. Indeed, we showed that invasive species might also have a higher seed production in nutrient‐poor soils if they invest less in plant size (Figure 6h). Interestingly, the interactions between traits and environment improved model explanatory power more for invasive than for noninvasive species (25% vs. 7%, Table 2), possibly indicating a higher flexibility to the environment in invasive than in noninvasive non‐natives. Related to these observations, we also report significant interactions between the habitat properties themselves, overall indicating high invader fitness in two distinct types of environments: (1) resource‐rich habitats, with higher temperatures, higher levels of soil nutrients, and higher diversity, yet lower light availability, and (2) resource‐poor habitats, with lower temperatures, lower soil nutrient levels, and lower diversity, yet higher light availability (Figures 4 and 5).

Biotic interactions have often been shown to be crucial (Mitchell et al., 2006) in regard to habitat invasibility. While abiotic conditions like climate undeniably play a primary role, especially on a larger spatial scale (Petitpierre et al., 2012), the local receptive plant community and its effect on the local abiotic environment defines the success of individual invaders (Levine, Adler, & Yelenik, 2004; Milbau et al., 2009). For example, we observed a fundamental role for native plant diversity of the receptive plant community in interaction with several plant traits. While diversity on its own did not explain patterns in seed production, low native species diversity did increase seed production in populations with large individuals of noninvasive species which had high foliar concentrations of trace elements (and low C/N‐ratios) or high levels of photosynthesis (Figure 7j–l). For invasive species, a positive effect of low native diversity was related to high seed production in populations with higher levels of photosynthesis or to a lesser degree in populations with smaller plants or plants with lower concentrations of trace elements (Figure 6j–l). These results support the often observed negative relationship of invasion with diversity on a local scale (Knight & Reich, 2005; Levine, 2000).

Biotic interactions can also be observed indirectly here through the role of light availability in the vegetation, which relates to aboveground competition. High light availability was indeed a good predictor of high seed production, especially if the non‐native population showed high photosynthetic efficiency (Figures 6, and 7d), or if they were tall (noninvasive species only, Figure 7e). This suggests that reduced aboveground competition can play an important role as driver of invasion success in our study system as well (Naeem et al., 2000), while the associated plant traits suggest a certain efficiency of these successful invaders, and thus probably competitive superiority in such environments.

Regardless of the observed patterns in seed production driven by habitat properties, plant traits, and their interactions, Figure 2 reminds us that species status plays a crucial role in explaining non‐native seed production (Hamilton et al., 2005; Mason, Cooke, Moles, & Leishman, 2008; Rejmanek & Richardson, 1996). Indeed, as the assessed invasive species in this study did not have a longer residence time than the noninvasive non‐natives, the observed discrepancy in seed production in our study system is likely part of the reason why the studied species became invasive or not (Moravcova, Pyšek, Jarošík, Havlíčková, & Zákravský, 2010). Interestingly, most single traits and properties related highly similarly to seed production in both noninvasive and invasive species, indicating that despite the overall differences in seed production, at first sight similar factors play a role as drivers of non‐native species fitness in these two distinct stages of the invasion process. The fact that invasive species in our study had a consistently higher seed production than their noninvasive counterparts with comparable trait values suggests that at least the traits used here on their own cannot be responsible for the successful seed production in the invasive species (Pysek et al., 2015). We did however observe additional variation in the direction of interactions between environmental properties and plant‐related traits for naturalized and invasive species (Figures 6 and 7), for example, with a more important role of plant size for naturalized than for invasive species in most environments (Figures 6 and 7 second column) and a stronger reaction of seed production to photosynthetic efficiency in invasive than in naturalized species (Figure 6 and 7 first column). Some of these different correlations could come from one universal nonlinear correlation, yet these results likely also suggest that the processes defining species naturalization might on a local scale—in interaction with the local environment—be different from those playing a role in the last stage of invasion (Pysek, Krivanek, & Jarosik, 2009; Richardson & Pysek, 2006), a conclusion strengthened by the observed stronger positive effect of trait × environment interactions on invasive than on noninvasive species (25% vs. 7%). We thus argue for an increased attention for the different stages of plant invasion (Milbau & Stout, 2008), in addition to the usual focus on invasive species only, the more common distinction between archaeophytes and neophytes or the assessment of all stages lumped together.

The detailed local measurement of all plant traits and habitat properties covered in this study is a resource‐intensive method, limiting the spatial extent and the size of the species set to which it could be applied. The resulting relatively small dataset inevitably hampers the explanatory power of our approach. Indeed, we could not assess three‐way interactions between variables, nor build one single model including all plant × environmental interactions, assess nonlinear relationships between factors, or investigate the subtle effects of single measurements (e.g., the photosynthetic efficiency in maximum light versus in shaded conditions or the different ecological role of above‐ and belowground plant size measures). Additionally, several habitat properties (like soil moisture or herbivory) and species characteristics (like seed dispersal characteristics or seed morphology, Pysek et al., 2015) with a proven effect on invader success were not taken into account. Yet we did manage to identify several important drivers of seed production for both noninvasive and invasive species that are often supported by other studies and ecologically meaningful interactions between those, even within the scope of our limited species set. The power of our approach thus truly lies in the general conclusion that an integrative approach combining both microscale environmental variables and population‐level trait variation, and several interactions among and between them, is needed to define invasion success at the local scale, more than in identifying widely applicable predictors of plant invader success.

Our detailed assessment of local traits and properties related to seed production resulted in several important take‐home messages that can improve our understanding of invader fitness on a local scale: (1) There is not one all‐encompassing driver of plant invasions that defines invader fitness: Even within the relatively small spatial scope of our study in Flanders, several habitat properties and species‐related traits played an undeniable role; (2) local measurements deserve much more attention than they currently get in plant invasion literature, yet (3) to truly understand plant invasions, this level of environmental and plant‐related detail will need to be combined with the generality of a multiregion and multispecies approach across different stages of invasion. It is thus time to work toward a unified approach in which databases of global species distribution data can be expanded with locally measured habitat properties and plant traits. Such a goal is ambitious, yet we believe it is achievable in the modern scientific landscape with its global consortia, international collaborations, and increasing attention for reproducibility of measurement protocols.

## AUTHOR CONTRIBUTIONS

ER and IN designed the experiment, ER gathered the data, JJL and ER performed the analyses, and JJL, ER, AM, and IN all contributed significantly to paper writing.

## CONFLICT OF INTEREST

None declared.
