# Supplementary material for: Habitat properties and plant traits interact as drivers of non‐native plant species’ seed production at the local scale
Source: Ecol Evol. 2018 Apr 2;8(8):4209–23. doi: 10.1002/ece3.3940 (PMC5916266; doi:10.1002/ece3.3940)
Supplement: Supplementary file 1 [file ECE3-8-4209-s001.docx]

***Table S1*** *PCA axis 1 and 2, and range of original values (minimum-mean-maximum) for each individual environmental measure within each of the 4 categories of habitat properties. Properties ordered based on their correlation with axis 1. When necessary, abbreviations are explained. For visualization of the first two axes of the PCAs, see Appendix Fig. 1(A-D)*

| **Temperature** | **Axis 1** | **Axis 2** | **Min-mean-max** | **Explanation** |
| --- | --- | --- | --- | --- |
| T_A_S | 0.921 | 0.144 | 16.4 - 24.9 - 38.9 °C | Temperature above vegetation – summer |
| T_B_S | 0.800 | 0.385 | 15.7 - 24.0 - 34.2 °C | Temperature below vegetation – summer |
| ΔT_S | 0.594 | -0.383 | -5 – 0.8 – 6 °C | Temperature difference – summer |
| T_A_L | 0.567 | -0.479 | 13.7 – 23.9 – 35.5 °C | Temperature above vegetation – spring |
| T_B_L | 0.486 | 0.406 | 14.7 – 22.7 – 30.6 °C | Temperature below vegetation – spring |
| ΔT_L | 0.146 | -0.947 | -6.8 – 1.2 – 13.8 °C | Temperature difference - spring |
| **Diversity** | **Axis 1** | **Axis 2** |  |  |
| R | -0.714 | 0.698 | 2 – 13.7 – 39 | Species richness |
| J | -0.909 | -0.375 | 0 – 0.8 – 2.0 | Pielou evenness index |
| H | -0.964 | -0.163 | 0 – 0.5 – 1 | Shannon diversity index |
| **Light** | **Axis 1** | **Axis 2** |  |  |
| PAR_A_S | 0.037 | -0.748 | 71 – 919.6 – 1640 μmol m^-^² s^-1^ | Photosynthetic active radiation above vegetation - summer |
| PAR_A_L | -0.069 | 0.665 | 94 – 970.1 – 1739 μmol m^-^² s^-1^ | Photosynthetic active radiation above vegetation - spring |
| PAR_B_S | -0.869 | -0.297 | 2 –223.6 – 1463 μmol m^-^² s^-1^ | Photosynthetic active radiation below vegetation - summer |
| PAR_B_L | -0.884 | 0.248 | 0 –213.3 – 1704 μmol m^-^² s^-1^ | Photosynthetic active radiation below vegetation - spring |
| PARr_L | -0.924 | -0.029 | 0.00 – 0.23 – 1.07 | Photosynthetic active radiation ratio - spring |
| PARr_S | -0.932 | -0.008 | 0.00 – 0.26 – 1.00 | Photosynthetic active radiation ratio - summer |
| **Soil nutrients** | **Axis 1** | **Axis 2** |  |  |
| Ca | 0.208 | -0.759 | 0.10 – 5.95 – 18.09 mg kg^-1^ |  |
| pH | 0.197 | -0.552 | 2.82 – 6.64 – 8.14 |  |
| Cu | -0.056 | -0.641 | 0 – 0.62 – 8.60 mg kg^-1^ |  |
| Mn | -0.092 | -0.638 | 2.60 – 26.90 – 186.60 mg kg^-1^ |  |
| Zn | -0.097 | -0.618 | 0.40 – 28.00 – 253.70 mg kg^-1^ |  |
| P | -0.113 | -0.430 | 0.30 – 23.78 – 148.70 mg kg^-1^ |  |
| Ccarbo | -0.164 | -0.373 | 0.00 – 3.48 – 23.93 % | Carbon in CaCO_3_ |
| Mg | -0.444 | -0.609 | 20.20 – 190.64 – 672.20 mg kg^-1^ |  |
| Al | -0.531 | 0.367 | 0 – 0.08 – 1.62 mg kg^-1^ |  |
| K | -0.593 | -0.091 | 6.80 – 148.69 – 752.20 mg kg^-1^ |  |
| Corg | -0.891 | 0.166 | 0.00 – 6.50 – 24.29 % | Organic carbon |
| Ntot | -0.906 | -0.060 | 0.00 – 0.34 – 1.37 % | Total nitrogen |
| Ctot | -0.942 | 0.034 | 0.40 – 6.54 – 25.78 % | Total carbon |

***Table S2*** *PCA axis 1 and 2 and range of original values (minimum-mean-maximum) for each individual species trait measure within each of the 3 categories of plant traits. Traits ordered based on their correlation with axis 1. When necessary, abbreviations are explained. For visualization of the first two axes of the PCAs, see Appendix Fig. 1(E-F)*

| **Photosynthesis** | **Axis 1** | **Axis 2** | **Min-mean-max** | **Explanation** |
| --- | --- | --- | --- | --- |
| Pmax_L | 0.855 | -0.177 | -1.19 – 13.71 – 46.50 μmol CO_2_ m^-^² s^-1^ | Light-saturated photosynthetic rate - spring |
| Rd_S | 0.803 | 0.066 | 0.06 – 0.93 – 3.10 μmol CO_2_ m^-^² s^-1^ | Dark respiration rate - spring |
| PARc_S | 0.769 | 0.037 | 0.39 – 13.02 – 51.47 μmol phot m^-^² s^-1^ | Light compensation point - summer |
| Pmax_S | 0.764 | -0.096 | 0.45 – 11.51 – 38.15 μmol CO_2_ m^-^² s^-1^ | Light-saturated photosynthetic rate - summer |
| α_L | 0.628 | 0.034 | 0.04 – 0.07 – 0.09 μmol CO_2_ μmol phot^-1^ | Apparent quantum efficiency - spring |
| PNUE_L | 0.598 | -0.527 | -15.6 – 155.8 – 412.1 μmol CO_2_ mol N^-1^ s^-1^ | Photosynthetic nitrogen use efficiency - spring |
| PNUE_S | 0.426 | -0.498 | 0 – 120.3 – 339.1 μmol CO_2_ mol N^-1^ s^-1^ | Photosynthetic nitrogen use efficiency - summer |
| PARc_L | 0.298 | 0.861 | -4.28 – 21.93 – 70.28 μmol phot m^-^² s^-1^ | Light compensation point - spring |
| α_S | -0.107 | -0.212 | 0.05 – 0.08 – 0.15 μmol CO_2_ μmol phot^-1^ | Apparent quantum efficiency - summer |
| Rd_L | -0.453 | -0.820 | -4.06 - -1.46 – 0.16 μmol CO_2_ m^-^² s^-1^ | Dark respiration rate - spring |
| **Plant size** | **Axis 1** | **Axis 2** |  |  |
| SLA_S | 0.698 | 0.248 | 68.95 – 330.68 – 1739.13 | Specific leaf area - summer |
| LMR_S | 0.663 | 0.336 | 0.09 – 0.33 – 0.70 | Leaf mass ratio - summer |
| SLA_L | 0.649 | 0.264 | 76.00 – 343.53 – 1857-59 | Specific leaf area - spring |
| LMR_L | 0.632 | 0.502 | 0.03 – 0.36 – 0.79 | Leaf mass ratio – spring |
| RSR_L | -0.328 | -0.522 | 0.04 – 0.47 – 4.37 | Root shoot ratio – spring |
| Br_S | -0.399 | -0.103 | 0.00 – 2.61 – 53.99 g | Root biomass – summer |
| Bt _S | -0.415 | 0.014 | 0.03 – 10.98 – 117.91 g | Total biomass – summer |
| Bt _L | -0.432 | -0.308 | 0.06 – 6.90 – 174.70 g | Total biomass – spring |
| Br_L | -0.435 | -0.394 | 0.01 – 2.27 – 62.20 g | Root biomass – spring |
| Height_S | -0.442 | 0.615 | 3 – 68 – 235 cm | Plant height – summer |
| RSR_S | -0.450 | -0.555 | 0.03 – 0.36 – 2.58 | Root shoot ratio – summer |
| Bl_L | -0.454 | 0.436 | 0.03 – 6.49 – 131.60 g | Leaf biomass – spring |
| Bs_L | -0.486 | 0.407 | 0.00 – 14.43 – 301 g | Shoot biomass – spring |
| Bs_S | -0.520 | 0.614 | 0.00 – 15.83 – 222.40 g | Shoot biomass – summer |
| Bl_S | -0.530 | 0.634 | 0.01 – 6.74 – 81.80 g | Leaf biomass – summer |
| Height_L | -0.546 | 0.592 | 5 – 56 – 235 cm | Plant height - spring |
| **Foliar chemical attributes** | **Axis 1** | **Axis 2** |  |  |
| CN_S | 0.729 | 0.589 | 6.94 – 18.16 – 43.31 | C/N ratio – summer |
| CN_L | 0.686 | 0.541 | 5.93 – 15.23 – 33.10 | C/N ratio – spring |
| C_S | 0.639 | -0.045 | 36.88 – 44.54 – 49.42 % | Carbon concentrations - summer |
| N_L | 0.184 | -0.464 | 0.27 – 1.35 – 3.24 % | Nitrogen – spring |
| Mn | 0.049 | 0.038 | 10.70 – 91.53 – 511.40 μg g^-1^ |  |
| Fe | -0.517 | -0.044 | 38.60 – 225.52 – 889.90 μg g^-1^ |  |
| Zn | -0.544 | 0.324 | 9.3 – 34.86 – 117.50 μg g^-1^ |  |
| K | -0.556 | 0.292 | 2224.8 – 11325.37 – 33115.00 μg g^-1^ |  |
| N_S | -0.600 | -0.658 | 0.00 – 1.41 – 4.68 % | Nitrogen concentrations – summer |
| Mg | -0.656 | 0.346 | 632.10 – 2402.14 – 7265.00 μg g^-1^ |  |
| Ca | -0.704 | 0.227 | 5611.80 – 20.944.02 – 75747.30 μg g^-1^ |  |
| P | -0.731 | 0.282 | 747.2 – 3263.02 – 7459.80 μg g^-1^ |  |
|  |  |  |  |  |
| Cu | -0.744 | 0.236 | 3.7 – 7.83 – 22.30 μg g^-1^ |  |

***Table S3*** *Estimates and P-values for the optimal model for the logarithm of seed production against the first axes of all PCAs of habitat properties and plant traits in interaction with species status (Status_N_ = non-invasive, as opposed to invasive). Stars indicate significant – and dots borderline significant – P-values. Optimal models derived from the full model without any two-way interaction between traits and properties after step-by-step removing the variable with the highest P-value and testing the effect of this removal with a likelihood test with P-value cut-off of 0.05*

|  | ***Estimate*** | ***P*** |
| --- | --- | --- |
| *(Intercept)* | *7.932* | *<0.001 ** |
| *Photosynthesis* | *0.122* | *0.222* |
| *Plant size* | *-0.205* | *0.258* |
| *Foliar chemical attributes* | *-0.091* | *0.359* |
| *Temperature* | *0.330* | *0.009 ** |
| *Light* | *-0.132* | *0.190* |
| *Soil nutrients* | *-0.270* | *0.050 ** |
| *Diversity* | *-0.019* | *0.837* |
| *Status_N_* | *-4.125* | *<0.001 ** |
| *Temperature:Status_N_* | *-0.407* | *0.068 .* |

***Table S4*** *Estimates and P-values for the optimal models for the logarithm of seed production against the first axes of all PCAs of habitat properties (top) and plant traits (bottom) in interaction with species status (Status_N_ = non-invasive, as opposed to invasive). Stars indicate significant – and dots borderline significant – P-values. Optimal models derived from the full models with all two-way interactions for properties and traits separately after step-by-step removing the variable with the highest P-value and testing the effect of this removal with a likelihood test with P-value cut-off of 0.025 (after Bonferroni-correction for multiple testing)*

|  | ***Estimate*** | ***P*** |
| --- | --- | --- |
| *(Intercept)* | *8.017* | *<0.001 ** |
| *Temperature* | *-0.074* | *0.464* |
| *Light* | *0.122* | *0.142* |
| *Soil nutrients* | *-0.221* | *0.059 .* |
| *Diversity* | *-0.012* | *0.833* |
| *Status_N_* | *-4.701* | *0.001 ** |
| *Temperature:Light* | *0.287* | *0.002 ** |
| *Temperature: Soil nutrients* | *-0.089* | *0.012 ** |
| *Light: Soil nutrients* | *-0.184* | *<0.001 ** |
| *Light:Diversity* | *-0.154* | *0.004 ** |
| *(Intercept)* | *8.180* | *<0.001 ** |
| *Photosynthesis* | *0.171* | *0.080 .* |
| *Plant size* | *-* | *-* |
| *Foliar chemical attributes* | *-* | *-* |
| *Status_N_* | *-4.539* | *<0.001 ** |

***Table S5*** *estimates and P-values for the optimal models for the logarithm of seed production against the first axes of all PCAs of habitat properties in interaction with photosynthesis (top), plant size (middle) and foliar chemical attributes (bottom) for invasive (left) and non-invasive (right) non-native species. Stars indicate significant – and dots borderline significant – P-values. Optimal models derived from the full model with all two-way interactions after step-by-step removing the variable with the highest P-value and testing the effect of this removal with a likelihood test with P-value cut-off of 0.016 (after Bonferroni-correction for multiple testing)*

|  | ***Invasive*** | | ***Non-invasive*** | |
| --- | --- | --- | --- | --- |
|  | ***Estimate*** | ***P*** | ***Estimate*** | ***P*** |
| *(Intercept)* | *0.328* | *<0.001 ** | *3.080* | *<0.001 ** |
| *Temperature* | *-3.646* | *<0.001 ** | *-2.500* | *<0.001 ** |
| *Light* | *17.456* | *<0.001 ** | *-0.149* | *0.080 .* |
| *Soil nutrients* | *-3.512* | *<0.001 ** | *-0.677* | *<0.001 ** |
| *Diversity* | *-2.349* | *<0.001 ** | *0.021* | *0.911* |
| ***Photosynthesis*** | *9.417* | *<0.001 ** | *0.729* | *<0.001 ** |
| *Temperature:Light* | *-* | *-* | *-0.202* | *<0.001 ** |
| *Temperature:Soil nutrients* | *1.788* | *<0.001 ** | *1.637* | *<0.001 ** |
| *Temperature:Diversity* | *3.807* | *<0.001 ** | *-1.339* | *0.003 ** |
| *Temperature:****Photosynthesis*** | *-4.651* | *<0.001 ** | *-* | *-* |
| *Light:Soil nutrients* | *8.869* | *<0.001 ** | *-* | *-* |
| *Light:Diversity* | *-5.610* | *<0.001 ** | *-1.591* | *<0.001 ** |
| *Light:****Photosynthesis*** | *-3.499* | *<0.001 ** | *-0.557* | *<0.001 ** |
| *Soil nutrients:Diversity* | *-8.518* | *<0.001 ** | *-* | *-* |
| *Soil nutrients:****Photosynthesis*** | *11.593* | *<0.001 ** | *0.203* | *<0.001 ** |
| *(Intercept)* | *10.350* | *<0.001 ** | *7.972* | *<0.001 ** |
| *Temperature* | *4.032* | *0.350* | *-4.184* | *<0.001 ** |
| *Light* | *-1.430* | *0.438* | *-3.229* | *<0.001 ** |
| *Soil nutrients* | *1.881* | *0.406* | *-2.698* | *<0.001 ** |
| *Diversity* | *1.869* | *0.459* | *1.132* | *0.048* |
| ***Plant size*** | *0.941* | *0.512* | *-1.366* | *<0.001 ** |
| *Temperature:Light* | *-2.450* | *0.415* | *2.147* | *<0.001 ** |
| *Temperature:Soil nutrients* | *-0.085* | *0.605* | *0.938* | *<0.001 ** |
| *Temperature:Diversity* | *-* | *-* | *-1.285* | *0.065* |
| *Temperature:* ***Plant size*** | *0.660* | *0.332* | *-* | *-* |
| *Light:Soil nutrients* | *-1.042* | *0.418* | *1.387* | *<0.001 ** |
| *Light:Diversity* | *-0.648* | *0.617* | *-2.174* | *<0.001 ** |
| *Light:****Plant size*** | *-* | *-* | *0.574* | *0.001 ** |
| *Soil nutrients:Diversity* | *0.650* | *0.239* | *-2.338* | *<0.001 ** |
| *Soil nutrients:****Plant size*** | *0.934* | *0.335* | *2.213* | *<0.001 ** |
| *(Intercept)* | *8.742* | *<0.001 ** | *4.283* | *<0.001 ** |
| *Temperature* | *0.858* | *0.003 ** | *-0.441* | *0.022 ** |
| *Light* | *-0.042* | *0.826* | *-0.525* | *<0.001 ** |
| *Soil nutrients* | *0.368* | *0.013 ** | *-1.220* | *<0.001 ** |
| *Diversity* | *0.033* | *0.760* | *0.059* | *0.767* |
| ***Foliar chemical attributes*** | *0.456* | *<0.001 ** | *-0.183* | *0.029 ** |
| *Temperature:Light* | *-0.780* | *0.005 ** | *0.285* | *0.101* |
| *Temperature:Soil nutrients* | *-0.173* | *0.002 ** | *0.197* | *0.002 ** |
| *Temperature:Diversity* | *0.234* | *0.004 ** | *-* | *-* |
| *Temperature:****Foliar chemical attributes*** | *-* | *-* | *-0.216* | *0.008 ** |
| *Light:Soil nutrients* | *-0.431* | *<0.001 ** | *-* | *-* |
| *Light:Diversity* | *-* | *-* | *-0.534* | *<0.001 ** |
| *Light:****Foliar chemical attributes*** | *-* | *-* | *-0.180* | *0.002 ** |
| *Soil nutrients:Diversity* | *-* | *-* | *-1.151* | *<0.001 ** |
| *Soil nutrients:****Foliar chemical attributes*** | *-* | *-* | *-* | *-* |
| *Diversity:****Foliar chemical attributes*** | *0.181* | *0.002 ** | *-* | *-* |

***
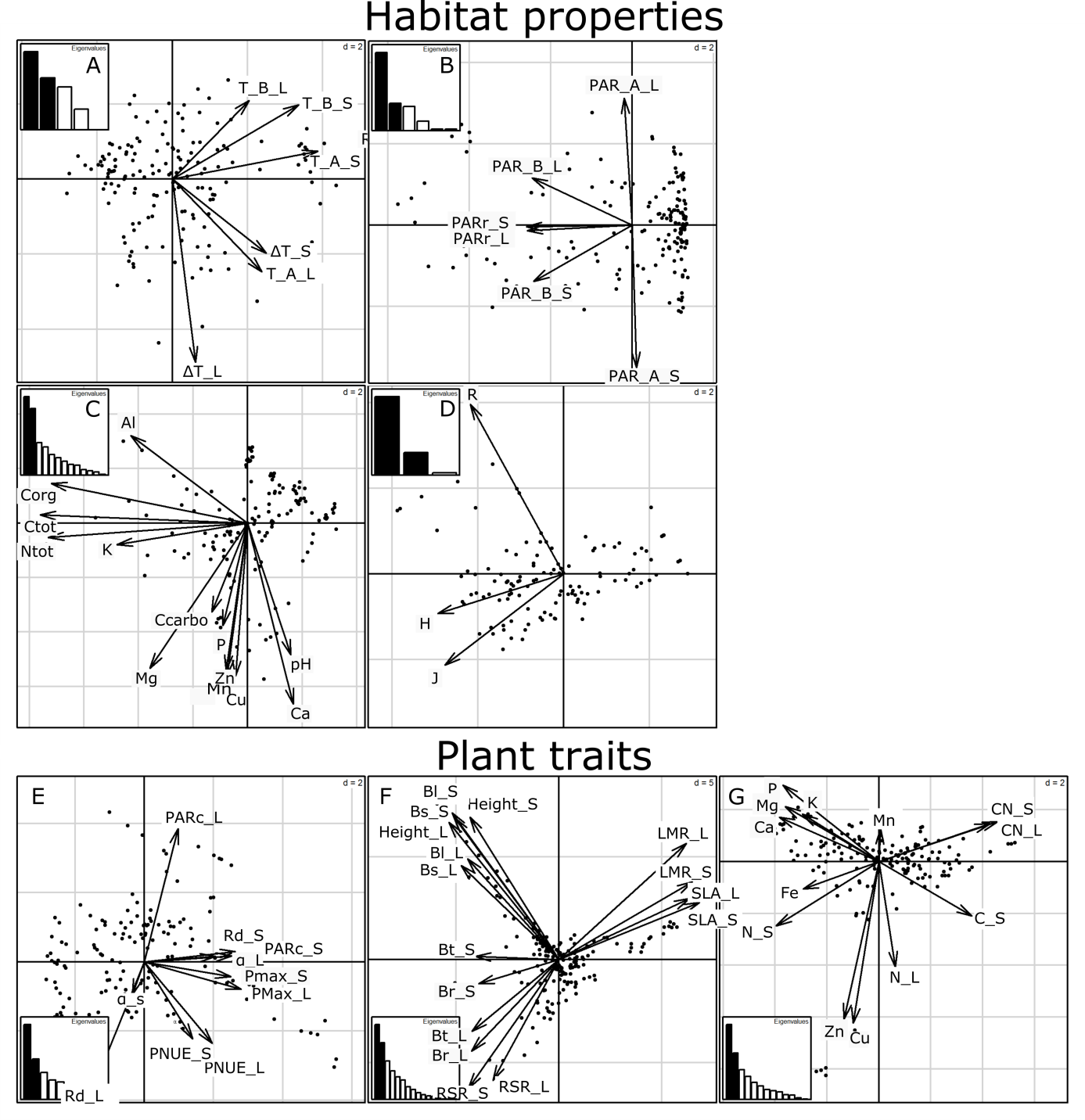
***

***Fig. S1*** *PCA axis 1 (x) and 2 (y) for the measures of four categories of habitat properties (A-D) and three categories of species traits (E-G): temperature (A), light (B), soil nutrients (C), native species diversity (D), photosynthesis-related traits (E), plant size (F) and foliar chemical attributes (G). Arrows indicate the relation of each individual measured habitat trait with both axes. The inset shows the relative importance of all PCA axes, with axis 1 and 2 in black. For the values for both axes for each measure, as well as the interpretation of the abbreviations, see Appendix Table 1 and 2*
